# Evaluation of a joint workshop on study design for hospital and community pharmacists: a retrospective cross-sectional survey

**DOI:** 10.1186/s40780-024-00337-x

**Published:** 2024-03-04

**Authors:** Yuki Asai, Yasushi Takai, Toshiki Murasaka, Tomohiro Miyake, Tomohisa Nakamura, Yoshihiko Morikawa, Yuji Nakagawa, Tatsuya Kanayama, Yasuharu Abe, Naoki Masuda, Yasushi Takamura, Yoshihiro Miki, Takuya Iwamoto

**Affiliations:** 1 Department of Pharmacy, Mie University Hospital, Faculty of Medicine, Mie University, 2-174 Edobashi, Tsu, Mie 514-8507 Japan; 2 Department of Pharmacy, Mie Heart Center Hospital, 2227-1 Ooyodo, Meiwa, Taki, Mie 515-0302 Japan; 3 Konan Pharmacy, 1874-4 Karasu, Tsu, Mie 514-0315 Japan; 4 Department of Pharmacy, Ise Red Cross Hospital, 1-471-2, Funae, Ise, Mie 516-8512 Japan; 5 Pharmacy, Mie Prefectural Mental Medical Center, 1-12-1, Shiroyama, Tsu, Mie 514-0818 Japan; 6 Ichishi Dispensing Pharmacy Takano Store, 226-7, Takano, Ichishi, Tsu, Mie 515-2504 Japan; 7 Sanai Pharmacy Ikuwa Store, 826-1, Daimon, Ikuwa, Yokkaichi, Mie 512-0911 Japan; 8 Mie Pharmaceutical Association, 311 Shimazaki, Tsu, Mie 514-0002 Japan

**Keywords:** Research activity, Community pharmacist, Hospital pharmacist, Study design, Workshop

## Abstract

**Background:**

Although pharmacists often identify numerous clinical questions, they face several barriers, including the lack of mentors for research activities in clinical settings. Therefore, a workshop for the appropriate selection of a study design, which is a fundamental first step, may be necessary. The purpose of this study was to evaluate the effectiveness of a workshop on study design for hospital and community pharmacists. Moreover, the characteristics of pharmacists with little involvement in research activities were extracted using decision-tree analysis to guide the design of future workshops.

**Methods:**

A workshop was conducted on October 1, 2023. It comprised three parts: lectures, group work, and presentations. Questionnaire-based surveys were conducted with workshop participants regarding their basic information, their background that influenced research activities, their satisfaction, and their knowledge/awareness. For the questions on knowledge/awareness, the same responses were requested before and after the workshop using a five-scale scoring system. Multivariate logistic regression analysis was conducted to identify independent factors influencing research activities. Decision tree analysis was performed to extract low-effort characteristics of the research activities.

**Results:**

Of the 40 workshop attendees, the overall satisfaction score for the workshop was 4.38 of 5, and the score for each question was 4 or higher. Significant increases were observed in the scores of knowledge/awareness after the workshop. Moreover, 95% of the pharmacists answered that it would be highly useful to conduct a joint workshop between hospitals and community pharmacists. Although independent influencing factors were not detected in the multivariate logistic regression analysis, the decision tree analysis revealed that pharmacists who were no member of an academic society (85%, 11/13) or members without any certifications or accreditations related to pharmacy practice (80%, 4/5) were the least active in clinical research. In contrast, those belonging to academic societies and holding certifications or accreditations related to pharmacy practice frequently conducted clinical research.

**Conclusion:**

The present study revealed that a joint workshop on study design may have the potential to change pharmacists’ knowledge and awareness of research activities. Moreover, future workshops should be conducted with pharmacists who do not belong to academic societies.

**Supplementary Information:**

The online version contains supplementary material available at 10.1186/s40780-024-00337-x.

## Background

Pharmacists often identify numerous clinical questions, and skills are required to conduct their own clinical research [1]. A recent survey at several Japanese national universities revealed that the number of papers increased from 2.87 per faculty member/year in the period from 1979 to 1980 to 10.77 per faculty member/year in the period from 2019 to 2020 at pharmaceutical and medical faculties [2]. This may reflect the increased research activity in the medical area at this educational institution. While research activities have been thriving in universities in which mentors are located, there may be barriers to research activities, such as the lack of mentors for clinical research in community pharmacies [3]. Therefore, it was considered necessary to establish a guidance system for research activities in clinical settings. Considering the current situation, the Mie Pharmaceutical Association established “The Research Activity Promotion Team” in February 2022 to promote clinical research activities by hospitals and community pharmacists.

The researchers selected a study design suitable for specific clinical questions, which is a fundamental first step [4]. In clinical research, it is necessary to understand the biases of each study’s design in terms of the subjects under investigation, such as related to the credibility of data, and to eliminate biases as much as possible to be able to appropriately interpret the results. The selection of an inappropriate design may potentially undermine the validity of clinical research [5], and it is thus important to conduct a workshop on how to select a correct research design. We held the first workshop on research design on October 2, 2022, for hospital and community pharmacists. One year after the first workshop, a second workshop was held and its effectiveness was evaluated. Furthermore, as research activities in the Mie Prefecture need to be stimulated, it is important to create an incentive for pharmacists to engage in clinical research, as they are commonly not involved in such research. It is thus necessary to elucidate the characteristics of pharmacists who are hardly involved in research activities to plan future workshops.

The purpose of the present study was to evaluate the effectiveness of the workshop based on changes in the participants’ knowledge and awareness of research activities. Moreover, the characteristics of pharmacists with little involvement in research activities were extracted using decision tree (DT) analysis to deduce the appropriate design of subsequent workshops.

## Methods

### Study design

We conducted a retrospective cross-sectional survey of 40 pharmacists, including hospital and community pharmacists, who attended the workshop. All participants were members of the Mie Pharmaceutical Association.

### Workshop

The workshop information was distributed via e-email to all pharmacies and hospitals belonging to the Mie Pharmaceutical Association in the period from August 21, 2023 to September 18, 2023. The workshop was held on October 1, 2023. The timetable is presented in Supplementary Table 1. First, the lectures focused on selecting an appropriate study design. Second, the research topics were assigned to two groups of five to six members each to construct the study project with the assigned responsible mentor. Finally, a presentation session was conducted to discuss the project.

### Questionnaire

Questionnaires were collected using Google Forms (Google, Mountain View, CA, USA). The questionnaire consisted of basic information, influencing backgrounds on research activities, satisfaction, and knowledge/awareness. Details of the questions are provided in Supplementary Table 2. For questions on knowledge/awareness, the same responses were obtained before and after the workshop. While questionnaires before the workshop were collected at the time of registration, a second questionnaire survey was performed immediately after the workshop.

### Outcome

The effectiveness of the present workshop was evaluated based on an increase in the participants’ knowledge and awareness of research activities via the questionnaires.

### Statistical analysis

While the Wilcoxon signed-rank test was used to examine the differences in continuous variables before and after the workshop, categorical factors were analyzed using McNemar’s test. Categorical variables were compared using the chi-square test or Fisher’s exact test.

For the question “Have you ever reported results of your research activities (conference presentations and/or research papers) since you started working? (Supplementary Table 2), “Only once or not at all” responses were defined as low involvement in research activities. For multivariate logistic regression analysis, low involvement was used as the objective variable and factors that exhibited *p* < 0.05 in the univariate analysis. DT analysis was performed according to our previous study [6], based on the chi-squared automatic interaction detection algorithm. All statistical analyses were performed using SPSS Statistics version 27 (IBM Japan, Tokyo, Japan), and the significance level was set at *p* < 0.05.

## Results

The questionnaire response rate was 100% (40/40 participants). The participating pharmacists had a wide range of experience, with more community than hospital pharmacists (Table 1).

**Table 1:** Background characteristics of participants

Factor	Responder, n	Rate, %^a^
Total	40	
Response rate	40	100
Age, years old		
20 to 29	9	23
30 to 39	12	30
40 to 49	9	23
50 to 59	7	18
60 to 69	2	5
≥ 70	1	3
Sex		
Male	26	65
Female	14	35
Workplace distribution		
Dispensing pharmacy	25	63
General Hospital / Clinic	15	38
Pharmacist experience, years		
< 1	2	5
2 to 5	6	15
6 to 10	8	20
11 to 20	11	28
21 to 30	10	25
≥ 31	3	8

^a^(Response / all responders) × 100

The overall satisfaction score for the workshop was 4.38, with all questions scoring 4 or higher (Table 2). While 75% of the respondents reported that time allotment was adequate, 20% desired more time for group work.

**Table 2:** Satisfaction level of the workshop participants

Question	Contents	Average Score^a^
1	Please indicate your overall level of satisfaction with the “workshop on study design”.	4.38
2	Did you understand the content of lecture?	4.40
3	Was the content of lecture appropriate for your skill?	4.53
4	Did you understand the content of group work?	4.48
5	Was the content of group work appropriate for your skill?	4.48
6	Have you become interested in research activities as a pharmacist?	4.28
7	How was the time allocated between the lecture and group work?	Response, n (%)^b^
	Appropriate	30 (75)
	Please increase the time of groupwork	8 (20)
	Please increase the time and content of lectures	2 (5)

^a^Respective score was calculated as 5-point scale for each question

^b^(Response / all responders) × 100

**Fig. 1:**
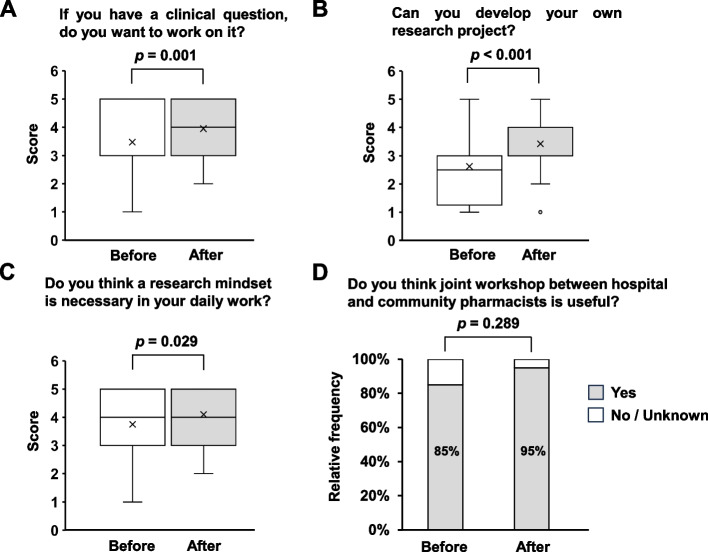
Workshop questionnaire on knowledge and awareness for research activities. **A** If you have a clinical question, do you want to work on it? **B** Can you develop your own research project? **C** Do you think a research mindset is necessary in your daily work? **D** Do you think that a joint workshop between hospitals and community pharmacists is useful?

As shown in Fig. 1A to C, significant increases in the respective scores were observed after the workshop. Although no statistically significant differences were observed, 95% of pharmacists answered that it would be highly useful to conduct another joint workshop (Fig. 1D). Moreover, respondents who answered “Yes” were most likely to comment “I would receive different opinions from different workplace distributions” (Supplementary Table 3).

Univariate analysis revealed that being a member of an academic society (*p* = 0.001, 95% confidence interval: 0.014‒0.427) and participating in the first study design workshop (*p* = 0.003, 95% confidence interval: 0.025‒0.525) were associated with clinical research efforts (Table 3). However, independent influencing factors for clinical research efforts were not detected in multivariate logistic regression analysis.

**Table 3:** Influencing factors for low efforts to research activities

Factors	Univariate analysis			Multivariate logistic regression analysis		
	OR	95%CI	*p* value	Adjusted OR	95%CI	*p* value
Sex	0.35	0.090–1.338	0.119^a^	-	-	-
Age (20 to 29 years old)	2.77	0.583–13.162	0.265^b^	-	-	-
Pharmacist experience (< 10 years)	1.18	0.333–4.195	0.796^a^	-	-	-
Workplace (dispensing pharmacy)	2.55	0.671–9.650	0.165^a^	-	-	-
Graduated from a six-year pharmacy school	1.18	0.333–4.195	0.796^a^	-	-	-
Presence of mentors in the workplace	0.53	0.150–1.880	0.324^a^	-	-	-
Obtained certifications or accreditations	0.23	0.050–1.052	0.049^a^	-	-	-
Membership in academic societies	0.08	0.014–0.427	0.001^a^	0.15	0.022–1.031	0.054
Participated in “the first study design workshop”	0.12	0.025–0.525	0.003^a^	0.28	0.049–1.558	0.145

*OR* Odds ratio, *95%CI* 95% coefficient interval

^a^Chi-square test

^b^Fisher’s exact test

**Fig. 2:**
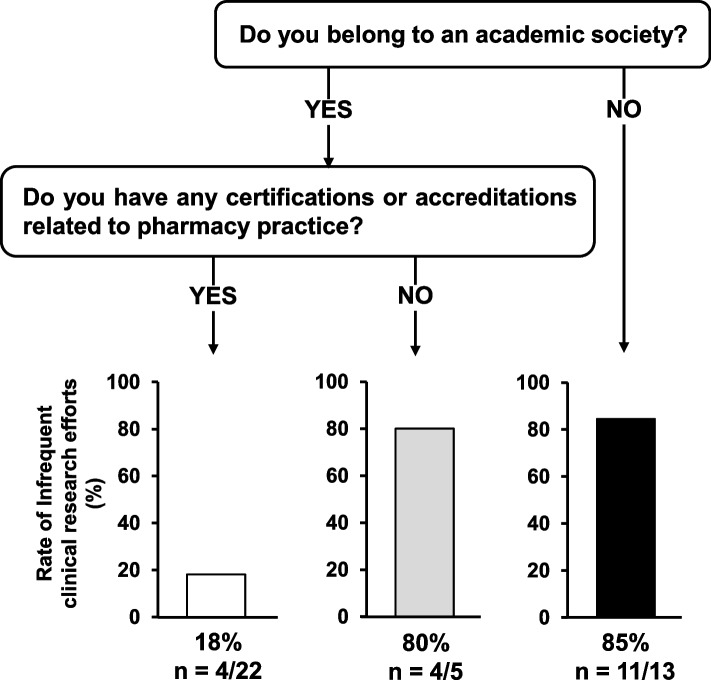
Decision tree model for predicting backgrounds of pharmacists with low involvement on research activities

In the DT analysis, pharmacists who did not belong to academic societies (85%, 11/13) or members who did not have any certifications or accreditations related to pharmacy practice (80%, 4/5) were hardly involved in clinical research, whereas those who were affiliated with academic societies and held certifications or accreditations were highly involved in clinical research (Fig. 2).

## Discussion

The questionnaire showed that the level of satisfaction was very high, and the content fit the needs of the participants. Moreover, an increase in knowledge and awareness of research activities was observed (Fig. 1), suggesting that the workshop was meaningful for pharmacists who want to start clinical research. E-learning and lectures have been reported as the most desired forms of learning in clinical research for community pharmacists [7]. Although the usefulness of face-to-face active workshops has been reported in clinical research learning methods [8], a concern was that more time for group work would result in fewer participants. However, no participants in this workshop desired a decrease in group work time; rather, some participants requested an increase (Table 2), indicating the need for group work and more opportunities for participants to discuss topics in depth in future workshops. The usefulness of this joint workshop was linked to a better understanding of the current situation regarding the different business categories and research activities of pharmacists in different professional fields (Supplementary Table 3).

Although the multivariate logistic regression analysis did not identify any independent factors associated with low involvement in research activities (Table 3), the DT analysis revealed that pharmacists who were not members of an academic society or members without any practical awareness may be associated with low involvement (Fig. 2). As membership is regularly required in academic societies, the results of the DT analysis may be as expected. Organizing workshops on more basic topics, such as the importance of research for pharmacists and how to identify clinical questions, may be of interest. Regular research conferences have been reported as a critical factor in research activities by resident physicians [9]. Moreover, as several reports have shown the usefulness of web-based educational programs received by community and hospital pharmacists [10, 11], web-based workshops may be useful depending on the level of achievement.

The present study has several limitations. First, as pharmacists who attended this workshop might have had a high awareness of research activities, the usefulness of this workshop might have been overestimated. Second, because the second questionnaire was performed immediately after the workshop, the results of this questionnaire might have yielded high ratings. Third, the factors influencing the low involvement in research activities may differ by population. Forth, the degree of the increase in the number of conference presentations and papers submitted by participants remains unknown.

## Conclusions

The present study revealed that a workshop on study design may have the potential to change pharmacists’ knowledge and awareness of research activities. Future workshops should target pharmacists who are not members of academic societies. Therefore, the Research Activity Promotion Team should continue to hold workshops and support research activities for the members of the Mie Pharmaceutical Association.

### Supplementary Information

**Additional file 1: Supplementary Table 1.** Contents of the workshop on study design. **Supplementary Table 2.** Questionnaire for the workshop. **Supplementary Table 3.** Reasons for the usefulness of joint workshops between hospitals and community pharmacists.

## Data Availability

The data supporting the findings of this study are available from the corresponding author upon reasonable request.

## Abbreviation

DT: Decision tree
