# Supplementary material for: Evaluation of a joint workshop on study design for hospital and community pharmacists: a retrospective cross-sectional survey
Source: J Pharm Health Care Sci. 2024 Mar 4;10:14. doi: 10.1186/s40780-024-00337-x (PMC10910793; doi:10.1186/s40780-024-00337-x)
Supplement: Supplementary file 1 — Additional file 1: Supplementary Table 1. Contents of the workshop on study design. Supplementary Table 2. Questionnaire for the workshop. Supplementary Table 3. Reasons for the usefulness of joint workshops between hospitals and community pharmacists. [file 40780_2024_337_MOESM1_ESM.docx]

**Supplementary Table 1 Contents of the workshop on study design.**

| Time schedule (P.M.) | Styles | Details |
| --- | --- | --- |
| 1:30-2:00 | Lecture | 1) Importance of conducting research by pharmacists  2) Needs to solve business improvement with a research mindset  3) How to select a research design  4) How to formulate a PECO/PICO  5) How to construct a study project |
| 2:00-2:50 | Group work | Formulate the clinical problem (target patients, exposure/intervention methods, etc.)  Theme 1: Hypertension  Theme 2: Polypharmacy  Theme 3: Oncology  Theme 4: Antipsychotic medications |
| 2:50-3:00 | Rest time |  |
| 3:00-4:15 | Group work | Formulate the clinical problem (comparators, outcome considerations)  Develop a research plan: consider feasible specifics |
| 4:15-4:30 | Presentation | One group presentation each study project |

**Supplementary Table 2 Questionnaire contents of the present workshop.**

|  | Contents |
| --- | --- |
| Basic information | |
| 1 | Age, years old |
|  | - 20 to 29, □ 30 to 39, □ 40 to 49, □ 50 to 59, □ 60 to 69, □ ≥ 70 |
| 2 | Sex |
|  | - Male, □ Female |
| 3 | Pharmacist experience, years |
|  | - < 1, □ 2 to 5, □ 6 to 10, □ 11 to 20, □ 21 to 30, □ ≥ 31 |
| 4 | Workplace distribution |
|  | - Dispensing pharmacy, □ General Hospital / Clinic |
| Influencing background for research activities | |
| 1 | Have you ever reported on the results of your research activities (conference presentations and/or paper publication) since you started working? If you answered “Yes”, how often? |
|  | □ At least once every year, □ About once every few years, □ Only once, □ Not at all |
| 2 | Did you get pharmacy license after pharmacy school transitioned to a six-year program? |
|  | - Yes, □ No |
| 3 | Are there a mentor for research activities in your workplace? |
|  | - Yes, □ No |
| 4 | Do you have any certifications or accreditations related to pharmacy practice? |
|  | - Yes, □ No |
| 5 | Do you belong to an academic society (except for the Japan Pharmaceutical Association and the Japanese Society of Hospital Pharmacists)? |
|  | - Yes, □ No |
| 6 | Did you attend the “the first workshop on study design"? |
|  | - Yes, □ No |
| Satisfaction | |
| 1 | Please indicate your overall level of satisfaction with the "workshop on study design". |
|  | Dissatisfaction←1 □, 2 □, 3 □, 4 □, 5 □→Satisfaction |
| 2 | Did you understand the content of the lecture? |
|  | Incomprehensible←1 □, 2 □, 3 □, 4 □, 5 □→Comprehensible |
| 3 | Was the content of the lecture appropriate for your skill? |
|  | Inappropriate←1 □, 2 □, 3 □, 4 □, 5 □→Appropriate |
| 4 | Did you understand the content of the group work? |
|  | Inappropriate←1 □, 2 □, 3 □, 4 □, 5 □→Appropriate |
| 5 | Was the content of the group work appropriate for your skill? |
|  | Inappropriate←1 □, 2 □, 3 □, 4 □, 5 □→Appropriate |
| 6 | Have you become interested in research activities as a pharmacist? |
|  | Not interested←1 □, 2 □, 3 □, 4 □, 5 □→Interested |
| 7 | How was the time allocated between the lecture and group work? |
|  | □ Appropriate, □ Please increase the time and content of lecture, □ Please reduce the time of lectures, □ Please increase the time of groupwork, □ Please reduce the time of group work, □ Others: |
| Knowledge/Awareness ^a^ | |
| 1 | If you have a clinical question, do you want to work on it? |
|  | I don't think so←1 □、2 □、3 □、4 □、5 □→I think so |
| 2 | Can you develop your own research project? |
|  | I don't think so←1 □、2 □、3 □、4 □、5 □→I think so |
| 3 | Do you think a research mindset is necessary for your daily work? |
|  | I don't think so←1 □、2 □、3 □、4 □、5 □→I think so |
| 4 | Do you think joint training between hospital and community pharmacists is useful? |
|  | □ Yes, □ No, □ Unknown |
|  | If you answered "Yes", please indicate the reasons. |
|  | □ I could receive different perspective opinions from different workplace distribution, □ I could receive the opinions of pharmacists with extensive research experience, □ I could understand the work situation of both community pharmacy and hospital, □ I could understand about the status of research activities in each workplace distribution, □ It would be beneficial to perform cooperation between community pharmacies and hospitals, □ Others: |

^a^ Responses to same questions were collected before and after the workshop.

**Supplementary Table 3 The reasons of usefulness for joint workshop between hospital and community pharmacists.**

| Reason | n^a^ |
| --- | --- |
| I could receive different perspective opinions from different workplace distribution. | 30 |
| I could understand the work situation of both community pharmacy and hospital. | 21 |
| It would be beneficial to perform cooperation between community pharmacies and hospitals. | 19 |
| I could receive the opinions of pharmacists with extensive research experience. | 16 |
| I could understand about the status of research activities in each workplace distribution. | 13 |

^a^Includes multiple-responses
